# Stacking Sequence Effect of Basalt/Carbon Hybrid Laminated Composites on Solid Particle Erosion Behavior: From Ambient to Elevated Temperatures

**DOI:** 10.3390/polym17101349

**Published:** 2025-05-15

**Authors:** Mehmet İskender Özsoy, Sinan Fidan, Mustafa Özgür Bora, Satılmış Ürgün

**Affiliations:** 1 Department of Mechanical Engineering, Faculty of Engineering, Sakarya University, Sakarya 54050, Turkey; 2 Faculty of Aeronautics and Astronautics, Department of Airframe & Powerplant Maintenance, Kocaeli University, Kocaeli 41001, Turkey; sfidan@kocaeli.edu.tr (S.F.); ozgur.bora@kocaeli.edu.tr (M.Ö.B.); 3 Faculty of Aeronautics and Astronautics, Department of Aviation Electrics and Electronics, Kocaeli University, Kocaeli 41001, Turkey; urgun@kocaeli.edu.tr

**Keywords:** hybrid composites, carbon fiber, basalt fiber, solid particle erosion, wear, high temperature

## Abstract

This is a research study on the high-temperature solid particle erosion behavior of basalt/carbon hybrid composites with varying ply arrangements (B_8_, C_8_, B_4_C_4_, C_4_B_4_, B_2_C_4_B_2_, and C_2_B_4_C_2_). Solid particle erosion experiments were carried out by employing garnet particles at temperatures of 25 °C, 50 °C, 80 °C, and 120 °C at impingement angles of 30° and 90°. The erosion weight loss rate differed substantially with the temperature, angle of impact, and ply arrangement. The highest erosion rates were obtained by single-component composites at 544.9 mg/g (B8, 120 °C, 30°) and 541.3 mg/g (C_8_, 120 °C, 90°). In contrast, the hybrid composites were more resistant, with the lowest rate being 200.0 mg/g at an ambient temperature (25 °C, 30°) for C_4_B_4_. The erosion weight loss at 50 °C increased typically due to thermal softening, whereas at elevated temperatures (80 °C, 120 °C), there was some stabilization seen, reflecting the positive synergies between basalt and carbon fibers. The factorial analysis of ANOVA revealed that material type (43.17%) was the most significant factor, followed by the temperature (19.97%) and impingement angle (0.52%). SEM and profilometry analysis confirmed that hybrid arrangements lower the erosion crater depth by a great extent, affirming the improved wear resistance of balanced basalt-carbon configurations. This work demonstrates the potential applications of optimally designed hybrid composites for durability under erosive high-temperature environments.

## 1. Introduction

In gas turbine engines, wind turbines, and hydroelectric power turbines, solid particle erosion (SPE) is a serious problem. SPE in gas turbine engines in aircraft increases fuel consumption and emissions, requires unscheduled removals, and increases maintenance costs. Moreover, it degrades operational readiness, safety, and mission success, which can lead to catastrophic failure during long flights in severe environments. Similarly, SPE is a severe problem for wind turbines, as the costliest components, namely fiber-reinforced polymer (FRP) composite blades, are severely eroded by rain [1]. Composite materials, mainly fiberglass reinforced with epoxy resins, have been used in the wind industry for decades. Because they operate in a severe environment, wind turbine blades are highly prone to erosion near the leading edge. Since surface wear is augmented by repeated exposure to airborne sand and hard particles, this problem is more pronounced near coastal areas for turbines installed there. This degradation not only complicates the process of maintaining the systems but also reduces the life of the blade, increases costs, and decreases the overall efficiency of wind energy systems [2]. Improving the wear resistance of composites is regarded as one of the most crucial issues to address in order to satisfy the design specifications of components when they are subjected to erosive environments. Erosion wear occurs whenever solid particles impinge on the surface of a material and cumulative damage is suffered by the target material. The target material density and hardness and operating parameters of the impact velocity, impingement angle, and erodent particle size and shape are important determinants of erosion. The fiber type and volume fraction have a pronounced effect on the erosion behavior in FRP composites [3]. FRP composites, in general, are less resistant to erosion compared to metallic materials; reinforced composites tend to suffer greater erosive wear compared to unreinforced polymer matrices. There are four main factors that affect the erosion rate of polymer composites: (a) target surface characteristics, the matrix material, reinforcement type and orientation, and matrix-reinforcement interface; (b) the test and environmental conditions, including temperature; (c) operating parameters, including the stand-off distance, impingement angle, velocity, and discharge rate; and (d) erodent characteristics, including the size, shape, type, and hardness. A number of interdependent properties need to be examined to establish the erosion behavior of solid particles [4]. Epoxy resins and other thermosetting resin-based composites have extensive applications in industries because of their low weight, high strength, and corrosion resistance [5,6,7,8,9]. Operating at temperatures up to 120 °C with very little dust, they are found to be quite effective in large shell structures for industrial gas exhaust ducts, minimizing abrasive effects on the inner surface of the duct. It is necessary to predict the duration for which these polymer composites would withstand high-temperature flue gases with abrasive environments. Hence, it is useful to investigate their gas abrasive wear resistance, particularly at high temperatures [10]. The influence of hybrid powder fillers on the thermal response and erosion resistance of woven-glass-fiber-reinforced epoxy composites was investigated. Erosion wear tests were carried out with alumina erodent at a 40 m/s velocity, 10 mm stand-off distance, and impingement angles of 30°, 60°, and 90° at ambient and elevated temperatures. Hybrid fillers enhanced the erosion resistance, with better performance compared to neat and single-filler composites. The samples exhibited brittle wear behavior at ambient temperature and a transition to slight semi-ductile behavior at high temperatures [11].

Nano-TiO_2_ and nanoclay fillers were examined to improve the erosive wear resistance of basalt-fiber-reinforced epoxy (BE) composites. Composites were formulated via vacuum-assisted resin infusion (VARI) and evaluated at different temperatures and impact velocities based on ASTM G76 standards. The erosion rates were found to be improved with velocity and temperature. Nano TiO_2_-filled composites were more resistant compared to nanoclay-filled and unfilled composites. Nano TiO_2_-reinforced samples showed lesser surface damage, as evidenced by scanning electron microscopy (SEM) analysis, suggesting improved erosion resistance [12]. Biswas and Satapathy [13] explored the erosion behavior of red-mud-filled glass-fiber-reinforced epoxy composites. Erosion wear tests were conducted using alumina erodent with a 40 m/s velocity, 10 mm stand-off distance, and impingement angles of 30°, 60°, and 90° under ambient and high temperatures. Hybrid fillers enhanced the erosion resistance and were found to be better than neat and single-filler composites. The samples exhibited brittle wear behavior at ambient temperature and a shift to minimal semi-ductile behavior at high temperatures. Padhi and Satapathy [14] explored the erosive behavior of epoxy bi-directional glass fiber composites filled with blast furnace slag. Four composites with 0, 10, 20, and 30 wt% slag were formulated by the hand layup technique, maintaining 40 wt% glass fiber as constant. All the samples showed semi-ductile behavior with maximum erosion at the impingement angle of 75°. Taguchi analysis validated the slag content, impact velocity, erodent temperature, and impingement angle as significant parameters. Artificial neural network analysis proved that 10 wt% slag-filled composites showed the lowest erosion rate. By the hand lay-up technique, an epoxy resin composite reinforced with glass fiber and aluminum nitride was prepared and characterized for its wear and erosion behavior. The tensile strength decreased with the filler content, whereas the hardness and erosion and wear resistance increased. Temperature had little effect on wear, whereas impact velocity was the most influential parameter, followed by the filler content and impingement angle. The significant parameters influencing erosion wear were established through Taguchi’s orthogonal arrays, and the material removal mechanism was established through SEM studies [15].

Mahapatra and Satapathy [16] developed an adaptive framework for polymer composite erosion performance prediction using statistical and machine learning (ML) models. Ramie–epoxy composites reinforced with varying contents (0–30 wt%) of sponge iron slag were prepared and tested for solid particle erosion at elevated temperatures according to Taguchi’s L27 orthogonal array. ANOVA revealed that filler content was the most dominating parameter (66.21%), followed by impact velocity (22.86%) and impingement angle (2.28%). A regression model and four ML models were developed for the prediction of erosion rates, among which the gradient boosting machine model showed the maximum accuracy and minimum errors. Jamali [17] investigated silanized graphene oxide (SGO)-reinforced basalt fiber/epoxy composites for their enhanced tribological and viscoelastic performance. Pin-on-disk wear tests and dynamic mechanical thermal analysis were performed for composites with various loadings (0–0.5 wt%) of SGO. The optimum 0.4 wt% SGO content was found to reduce the wear rate and friction coefficient by 62% and 44%, respectively, compared to a neat basalt/epoxy composite. Moreover, the 0.4 wt% SGO composites enhanced the storage modulus by 130% and glass transition temperature by 13.6 °C. A polymer matrix composite reinforced with unidirectional AS4 carbon fiber was subjected to solid particle erosion. In this study, 100 μm sieved runway sand particles and 10 μm Arizona road dust particles were used in the erosion tests, which were carried out at temperatures up to 260 °C and impact velocities up to 152.4 m/s. The results show quasi-ductile erosion behavior, with a 45° impact angle displaying the maximum erosion rate. Road dust erosion was found to be more than doubled by sand, and the erosion rates were found to increase with temperature and impact velocity. Erosion depended on the fiber orientation, with lower rates at 260 °C and higher rates for 90° orientation at room temperature [18]. Wu et al. [19] investigated how the glass-to-rubber transition of the resin matrix influences the friction and wear characteristics of friction materials, considering various types of thermosetting resins. The coefficient of friction (COF) values increased as the temperature rose from 100 to 200–250 °C but decreased beyond 300 °C due to adhesive resin degradation, weakening mechanical strength, wear resistance, and friction coefficient. The resin remained in the glassy state at 100 °C, restricting the movement of polymer chains and matrix deformation. Composites with a greater storage modulus had a lower COF due to the fact that there was less real contact area under loading. On the contrary, lower storage modulus composites deformed more, leading to a larger real contact area and friction force, and higher COF values of friction pairs. Pihtili [20] examined the impact of resin content on the wear behavior of woven roving glass fiber–epoxy resin and glass fiber–polyester resin composites. Surface temperature is a crucial factor in analyzing the tribological behavior of polymer composites; however, it has received limited attention. The high stiffness and low thermal conductivity in most polymer composites lead to elevated sliding contact temperatures, causing a sharp increase in wear rates beyond a critical threshold. The weight loss of plain polyester resin increased after a sliding distance of 942 m due to rising temperatures. In contrast, the wear on glass fiber–polyester composites decreased as higher temperatures facilitated material removal from the surface. The erosion tests of polymer composites are mostly conducted at room temperature (RT), as in Ref. [21]. Polymers with a glass transition temperature (Tg) above RT undergo higher erosion, while those with Tg below RT undergo lesser wear. For heated solid particles, the target material loses thermal along with kinetic energy, and the surface undergoes severe damage. The erosion rates were reported to rise with temperature for stainless steel, as was observed for epoxy polymer composites.

Hybridization was found to improve the mechanical behavior of composites, especially where the addition of fillers or fibers in polymer composites resulted in improved flexural, tensile and impact strength [22,23,24,25,26]. Furthermore, hybridization can also improve the tribological behavior of fiber-reinforced polymer composites, where laminate stacking sequences, operating conditions, and material parameters all influence wear behavior. By modeling desirable responses, response surface methodology can also optimize wear resistance through optimizing parameters such as the sliding distance and applied stress [27,28]. Saroj and Nayak [29] reported the effect of carbon and glass fiber hybridization with flax and kenaf fibers on the properties of a tribo-mechanical hybrid composite. Amongst the composites fabricated, flax-carbon (C_2_F_3_C_2_) exhibited the maximum flexural strength (364.4 ± 15.0 MPa) and impact resistance, while kenaf-glass (G_2_K_3_G_2_) was the best in terms of wear resistance. Sanman et al. [30] investigated the abrasive wear behavior of natural-fiber-reinforced hybrid polymer matrix composites on a pin-on-disc wear test rig. Abaca, basalt, and hybrid abaca–basalt-fiber-reinforced epoxy composites were subjected to an L9 orthogonal array for varying load, speed, and time conditions on 320 µm silicon carbide paper. The results indicated that basalt fiber composites experience minimum weight loss at 10 N, 200 rpm, and 10 min, and that abaca fiber composites experience minimum weight loss at 30 N, 300 rpm, and 20 min. Material composition is found to be the most important factor affecting wear resistance. Sathish et al. [31] reported the fabrication of a hybrid polymer matrix composite by flax and hemp fiber loops in an epoxy matrix. The composites were subjected to tribological and mechanical properties, utilizing the Taguchi L16 orthogonal array for optimizing the sample preparation. The compression molding parameters, namely the reinforcement percentage (20–50%), molding temperature (150–180 °C), pressure (1–4 MPa), and curing time (20–35 min), were optimized. It was observed that the reinforcement percentage influences the fatigue strength and that the curing time influences the impact resistance and wear behavior significantly, enhancing the overall composite performance. Antil et al. [32] reported the bonding behavior of S-type woven-glass-fiber-reinforced polymer matrix composites (PMCs) in natural abrasive slurry environments. Response surface methodology (RSM) was employed to investigate the effect of the slurry pressure, impingement angle, and nozzle diameter on the erosion resistance, with erosion loss as a response factor. Artificial neural network (ANN) modeling was used for validation and optimization. A comparative analysis between the RSM and ANN models indicated close agreement, confirming their effectiveness in predicting the erosion behavior of glass-fiber-reinforced PMCs. Mahapatra and Satapathy [33] discussed the erosion performance of titania (TiO_2_)-filled ramie–epoxy hybrid composites using artificial neural networks (ANNs) and statistical analysis. The composites, which were prepared by hand lay-up, were tested for solid particle erosion following Taguchi’s L27 orthogonal array. The impact velocity and filler content had pronounced influences on the erosion rate. Steady-state erosion tests provided individual factor influences. Erosion rates were predicted by an ANN model with 90% accuracy, with relative errors in the range 1–10%. Validation with steady-state erosion data gave evidence of the model’s reliability, proving its utility for the prediction of composite erosion behavior.

In the current investigation, solid particle erosion experiments were conducted on hybrid composites with six different ply sequences (B_8_, C_8_, C_4_B_4_, B_4_C_4_, C_2_B_4_C_2_, B_2_C_4_B_2_) at four different temperatures (25 °C, 50 °C, 80 °C, and 120 °C) and two impingement angles (30° and 90°) using garnet as the abrasion medium. Unlike previous research efforts that have widely been concerned with the erosion response of polymer matrix composites (PMCs) or hybrid composites at room temperature, the current research is unique as it investigates the influence of high temperatures on the solid particle erosion resistance of basalt/carbon hybrid laminates. While an extensive amount of research has been dedicated to the investigation of the erosion resistance of carbon-fiber-reinforced and basalt fiber-reinforced composites separately, the synergistic effects of their hybridization under different thermal conditions have not been investigated to a significant degree. Furthermore, the effect of the ply sequence on erosion resistance at high temperatures is a crucial aspect that has not been thoroughly investigated in the literature. The current research aims to bridge this knowledge gap by examining the influence of different stacking configurations on the erosion behavior of hybrid composites by taking into account the thermal softening of the matrix as well as the fiber–matrix interfacial integrity at elevated temperatures. The findings of this research will provide important insights into the viability of utilizing basalt/carbon hybrid laminates in high-temperature erosive conditions, which will contribute to designing and developing more erosion-resistant composite materials for demanding engineering applications.

## 2. Materials and Methods

### 2.1. Materials and Manufacturing of the Composites

**Figure 1:**
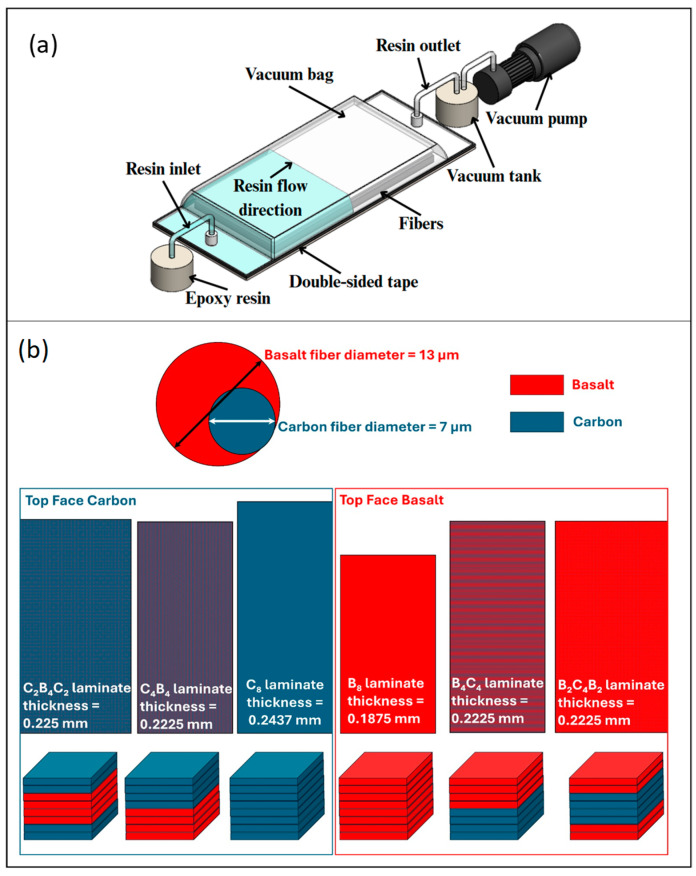
(**a**) Vacuum infusion method, (**b**) fiber diameters, laminate sequences and laminate thickness of the composites.

Two-component epoxy resin (Sika CR80 epoxy and CH80-2 hardener), used as the matrix material, was provided by Tekno Endustriyel Kimyasallar Corp., Istanbul, Turkiye. This epoxy resin has a density of 1.01 g/mL and a tensile strength and tensile modulus of 83 MPa and 2900 MPa, respectively. Its Shore D hardness is 84 and glass transition temperature is 93 °C. Carbon and basalt woven fabric were supplied by Dost chemical corporation, Istanbul, Turkiye. These two fabrics both have 200 gr/m^2^ areal densities. The carbon fiber has a tensile strength of 3950 MPa and elastic modulus of 238 GPa. Its density is 1.76 g/cm^3^ and its thermal conductivity and coefficient of thermal expansion are 17 W/m.K and −0.1 × 10^−6^/°C, respectively. The basalt fibers exhibit a tensile strength of about 3100 MPa and an elasticity modulus ranging from 88 to 92 GPa. Its density is about 2.60 to 2.63 g/cm^3^. The thermal conductivity and coefficient of thermal expansion of basalt fibers are about 0.031–0.038 W/m.K and 8 × 10^−6^/°C, respectively, according to supplier data sheets. Composites were manufactured as B_8_, C_8_, C_4_B_4_, B_4_C_4_, C_2_B_4_C_2_ and B_2_C_4_B_2_ sequences by the vacuum infusion method with 400 × 400 mm^2^ dimensions (Figure 1a). Once the composites had been cured at room temperature for 24 h, they were subjected to post-curing at 60 °C for 4 h. Then, composite plates were cut to sample sizes with a water jet machine. Figure 1b shows the fiber sequences of the laminate composites.

### 2.2. Solid Particle Erosion Tests

**Figure 2:**
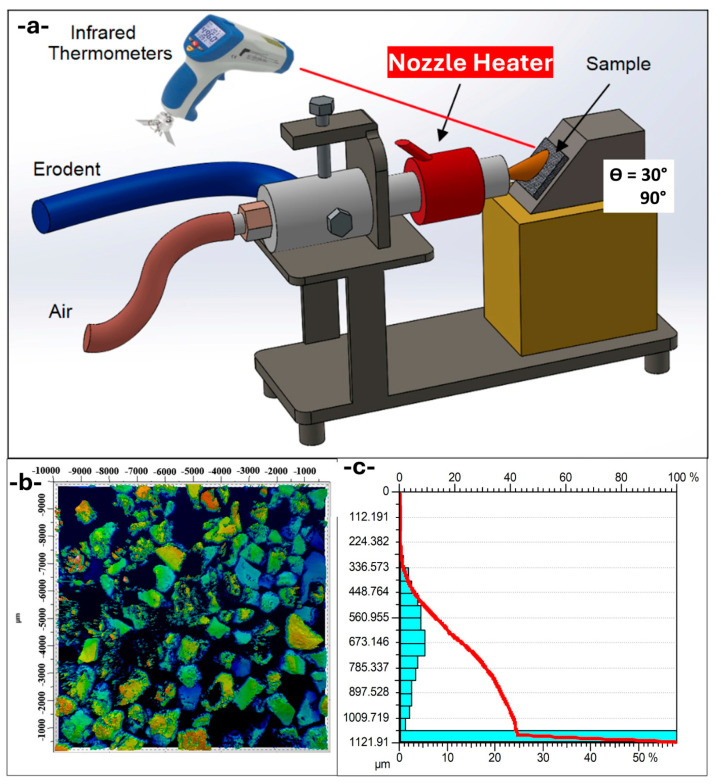
(**a**) Schematic of high-temperature solid particle erosion test set-up, (**b**) abrasive garnet particles; (**c**) abrasive garnet particle size distribution histogram.

The abrasive garnet particles used in this study possess a well-known chemical composition and physical properties that destine them to be used in various industrial fields, most commonly in cutting, sandblasting, and waterjet machining. Abrasive garnet particles contain high contents of silicon dioxide (SiO_2_) (34–40%) and aluminum oxide (Al_2_O_3_) (17–25%), which account for their high hardness and durability. Iron oxides (FeO + Fe_2_O_3_) (28–31%) account for their abrasion efficiency, while magnesium oxide (MgO) (5–8%) and small quantities of manganese oxide (MnO), titanium dioxide (TiO_2_), and calcium oxide (CaO) also play a role in their mechanical properties. Their bulk density ranges from 2.1 to 2.4 g/cm^3^, and their specific gravity ranges from 4.0 to 4.2 g/cm^3^, indicating their relatively high mass per unit volume, which is advantageous in abrasive blasting operations. Also, their 7.5–8 hardness on the Mohs scale indicates their ability to abrade and cut hard materials with efficiency. The particle erosion tests were carried out by blasting garnet abrasive particles onto the surfaces of the target composite materials using compressed air in a specially designed test chamber, following the specifications of ASTM G76 standards (Figure 2a) [34]. The solid particle erosion tests were performed at 25 °C (room temperature), 50 °C, 80 °C, and 120 °C. During the investigations, three iterations were performed for each material category at each angle, and the average mass losses of the target materials were calculated. The margin of error, calculated from a series of iterations of trials, was 2.75%. To determine the reliability of the data, a relative standard deviation (RSD) for every set of triplicate tests was calculated, which proved to be consistently less than 2.75% throughout. The number represents the highest deviation from the average for a set of groups and demonstrates reproducibility and accuracy within the experiments performed for erosion measurement.

Before and during the erosion testing process, the specimens were weighed using a high-precision scale with an accuracy of 0.1 mg (Shimadzu ATX-224, Kyoto, Japan). The erosion rate was determined by dividing the mass loss (Δm) of the target material layered composites during testing by the total weight of the abrasive ceramic bead and garnet particles (M) used in the experiment. The erosion rate calculation is expressed in Equation (1).

(1) $$\mathrm{Erosion}\mathrm{Rate}\;=\;\frac{\Delta m}{M}$$

The erodent utilized in the experiment consists of garnet. The size of the garnet abrasive particles ranged from 400 to 1150 μm (Figure 2b,c). The particle impingement angles were set at two distinct values: 30° and 90°. The experiment operated using a 5 mm nozzle diameter and 2 bar acceleration/blast gun pressure. The garnet erodent stream was operated at a mass flow rate of 22.5 g/s and a velocity of 58 m/s. While the impingement velocity remained a constant 58 m/s based on the specifications of ASTM G76 regarding the maintenance of controlled testing conditions, future studies would be able to ascertain the synergistic effects of velocity alongside the temperature and architecture of materials to more clearly reveal the entire erosion response of composite hybrids. Under regulated temperatures of 25 °C, 50 °C, 80 °C, and 120 °C, the experiments allowed a ±2 °C variance. Each test had a duration of 10 s and was carried out at a distance of 20 mm. Abrasive speeds were measured at the nozzle exit and by the double disk speed measurement method, the details of which are described in the literature [35].

### 2.3. Erosion Crater Depth Analysis

The size of the erosion craters was evaluated using a non-contact laser profilometer after a solid particle erosion test. Surface erosion craters were evaluated using a Nanovea PS50 non-contact laser profilometer. Unprocessed images taken during the process were processed using DigitalSurf software by Mountains Technology, version 6.2.7487, to produce three-dimensional surface topography images based on the ISO 25178 standard [36]. These essential photos were generated using the erosion crater data to ascertain their depth according to ISO 4287 [37]. The laser profilometer facilitated a comprehensive investigation of the erosion crater’s surface by scanning samples measuring 15 mm × 15 mm. The depth was assessed by measuring from the center of the erosion crater to the point where the abrasive jet impacted perpendicularly. Using scanning electron microscopy (SEM), the erosion damage mechanisms at the erosion crater of the composite materials were identified. Scanning electron microscopy, JEOL (JSM-6060LV, Tokyo, Japan) facilitated the examination of erosion damage on the composite material’s surface.

## 3. Results

### 3.1. Solid Particle Erosion Results

**Figure 3:**
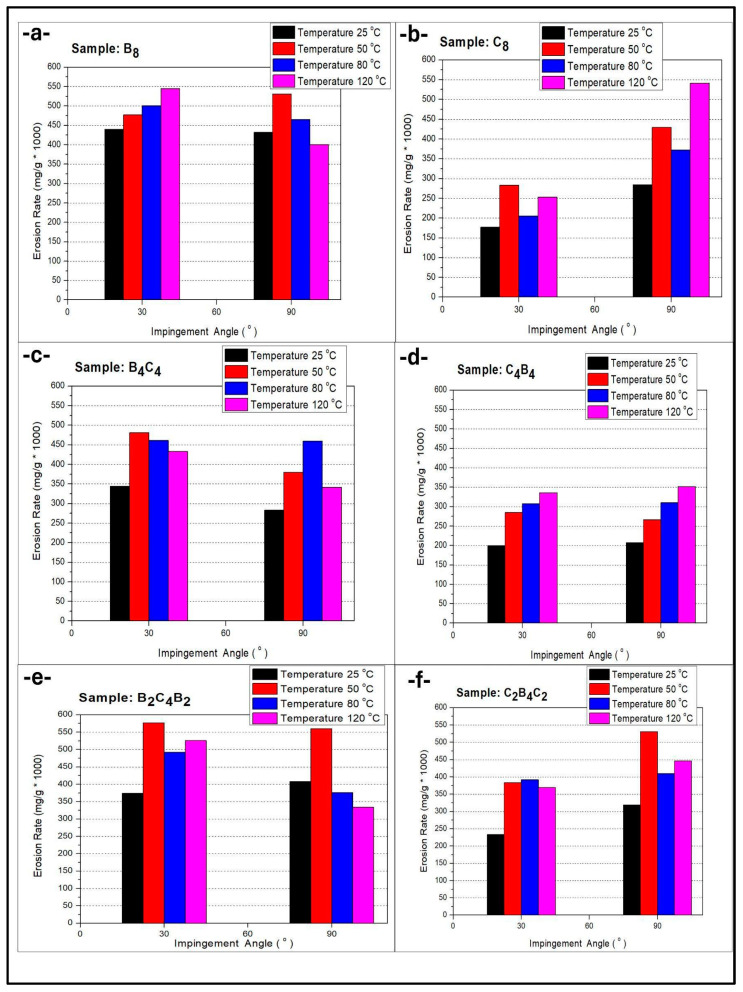
Erosion rate comparison at 30° and 90° impingement angles with various temperatures: (**a**) B_8_, (**b**) C_8_, (**c**) B_4_C_4_, (**d**) C_4_B_4_, (**e**) B_2_C_4_B_2_, (**f**) C_2_B_4_C_2_ (Error: 2.75%).

Figure 3a shows the erosion rates of the B_8_ sample at different temperatures and different impact angles. In the graph, it is observed that there is a significant increase in the erosion rate as the temperature increases. At the 90° impact angle, the highest erosion rate was obtained at 120 °C. This situation shows that high temperatures can negatively affect the wear resistance of basalt-based materials. Figure 3b presents the erosion rates of the C_8_ sample. The effect of temperature is significant for this carbon-based material, and a sharp increase in the erosion rate is observed, especially at 120 °C. While lower wear rates are observed at low temperatures, it can be said that as the temperature increases, the structural integrity of the material decreases and it becomes more susceptible to wear. While rising temperatures up to a moderate level can soften and harden partially, making the epoxy matrix tougher and more ductile, and hence more crack-absorbing, the overall erosion behavior of fiber-reinforced boards at high temperatures is controlled by a more complicated interaction. Over a given threshold, say 80 °C or 120 °C for our studies, the epoxy matrix reaches or exceeds the glass transition temperature (Tg), which increases the loss of mechanical stiffness and interface strength. Thermal degradation loosens fiber–matrix adhesion and increases debonding, which deteriorates the capacity of the material to absorb impact load. Moreover, any thermo-oxidation of the surface could further degrade mechanical integrity. Consequently, while minimal toughening would slow erosion at an intermediate temperature (for instance, 50 °C), greater thermal exposure would enhance erosion due to matrix softening, deteriorated interfaces, and a diminished load-carrying capacity. Figure 3c shows the erosion behavior of the B_4_C_4_ composite sample. This structure, which contains both basalt and carbon, exhibits a moderate sensitivity to temperature. While the highest erosion rate is observed at 50 °C, a significant decrease is observed at higher temperatures. This situation suggests that certain synergistic effects come into play as the temperature increases and that the thermal stability of the material may have increased. Figure 3d analyzes the wear behavior of the C_4_B_4_ composite material. An increase in the erosion rate is observed as the temperature increases, but this increase is more controlled compared to the B_8_ and C_8_ samples. This result shows that the combination of basalt and carbon in a balanced ratio can make a positive contribution to the wear resistance. Figure 3e presents the erosion rates of the B_2_C_4_B_2_ composite material. The highest wear rate was observed at 50 °C, which shows that the material is more prone to wear in certain temperature ranges. The decrease in the erosion rate at 80 °C and 120 °C indicates that the bonds in the material can be stabilized at high temperatures. Figure 3f shows the erosion rates of the C_2_B_4_C_2_ sample. It exhibits a more stable behavior against temperature changes compared to other composites. Although there are no major changes at different impact angles, a slight increase in the erosion rate is observed as the temperature increases. This result suggests that the combination of carbon and basalt in certain proportions can increase the resistance to temperature changes.

When Figure 3 is evaluated, it is seen that the effect of the layer arrangement on the erosion rate is directly related to the temperature and impact angle. The B_8_ and C_8_ samples consisting of a single type of layer show the highest erosion rates, especially at high temperatures, suggesting that homogeneous basalt or carbon structures are more vulnerable to abrasion. It is noteworthy that the effect of temperature becomes more pronounced as the carbon content increases, and a sharp increase is observed at 120 °C in the C_8_ sample. In contrast, in composites such as B_4_C_4_ and C_4_B_4_, which contain a combination of basalt and carbon layers in certain proportions, the erosion rate is relatively more balanced. This shows that the synergy formed by the combined use of basalt and carbon can increase the abrasion resistance of the material. The B_2_C_4_B_2_ and C_2_B_4_C_2_ samples with more complex layer arrangements exhibited more stable abrasion behavior against temperature increases, and the changes in the erosion rate remained at lower levels. While there is a significant increase in erosion especially at 50 °C, the decrease in erosion rate at high temperatures indicates that these layer combinations can be more resistant at certain temperature ranges. In general, it can be stated that mixed layer structures, and especially balanced basalt and carbon combinations, exhibit a structure that is more resistant to erosion and are less sensitive to temperature changes. In Figure 3, it is observed that the effect of temperature on the erosion rate varies depending on both the material composition and the impact angle. While relatively low erosion rates are observed for all samples at 25 °C, a significant increase in wear rates occurred, especially in the B_8_ and C_8_ samples, with homogeneous layer structures as the temperature increased. While the highest wear rate was observed in some composite samples (e.g., B_2_C_4_B_2_) at 50 °C, it can be said that a weakening of the mechanical properties of the materials occurred at this temperature and that the resistance to wear decreased. As the temperature increased to 80 °C and 120 °C, a relatively more stable route was observed in the erosion rates in some of the samples (e.g., C_2_B_4_C_2_); however, in some, lower wear rates were observed compared to 50 °C. This suggests that some of the factors that may occur at the surface, such as the stabilization of bonds within the material at a high temperature or oxidation, can improve wear resistance. In particular, the combination of carbon and basalt layers in certain proportions provides more stable wear resistance by balancing the changes in the erosion rate when the temperature increases. Therefore, it can be concluded that while the effect of temperature is more pronounced in single-type materials, this effect varies depending on the internal structure of the material in multi-layered and composite structures. More extreme angles such as 45° and 60° were considered, yet for this initial investigation, only the comparative extremes were examined (30° and 90°) to allow for the isolation of the dominant erosion processes, namely cutting and deformation, and the assessment of different thermal and material conditions. Subsequent studies will include intermediate angles for determining the more detailed mapping of the transitioning behavior.

### 3.2. Erosion Crater Depth and Roughness Characterization

**Figure 4:**
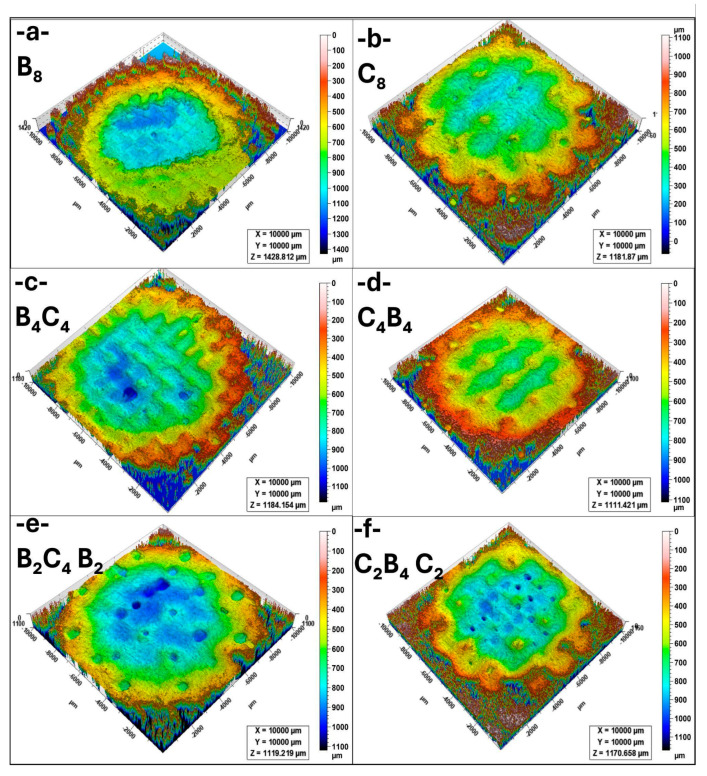
Comparison of 3D images of erosion crater at 90° impingement angle and 25 °C (**a**) B_8_, (**b**) C_8_, (**c**) B_4_C_4_, (**d**) C_4_B_4_, (**e**) B_2_C_4_B_2_, (**f**) C_2_B_4_C_2_.

Figure 4a presents the erosion crater of the B_8_ sample. The crater exhibits a deep central region with relatively steep edges, indicating a high material removal rate. The erosion depth suggests that the homogeneous basalt structure is more vulnerable to material loss under normal impingement conditions. The significant material displacement at the center is consistent with the high erosion rates observed in Figure 3, implying that the brittle nature of basalt contributes to severe surface degradation. Figure 4b illustrates the erosion crater of the C_8_ sample. Compared to B_8_, the erosion appears more uniformly distributed across the surface, though the depth remains substantial. The carbon-based structure demonstrates high erosion susceptibility, particularly at the impact center. The crater edges show a rougher transition, suggesting that the carbon material undergoes fragmentation rather than cohesive material loss. This aligns with the high erosion rates reported for C_8_ in Figure 3, reinforcing the idea that pure carbon structures are prone to significant erosion. Figure 4c displays the B_4_C_4_ sample, which consists of alternating basalt and carbon layers. The crater depth is shallower than in B_8_ and C_8_, indicating improved erosion resistance. The surface morphology exhibits a more dispersed erosion pattern, suggesting that the composite structure enhances material durability by distributing impact stresses more evenly. This confirms the protective effect of layer alternation in reducing the overall material removal rate. Figure 4d shows the erosion crater of the C_4_B_4_ sample. Similar to B_4_C_4_, this configuration results in a shallower crater than the single-material structures. However, the erosion distribution appears less uniform, with some localized deeper regions. This may be due to differences in the way carbon and basalt layers interact under erosion conditions. The improved performance compared to homogeneous samples suggests that the composite structure enhances resistance to impact damage. Figure 4e presents the erosion crater of the B_2_C_4_B_2_ sample. The crater depth is significantly reduced compared to single-material samples, demonstrating enhanced erosion resistance. The erosion morphology is more complex, with multiple small erosion pits instead of a single deep cavity. This suggests that the increased layering of basalt and carbon contributes to better stress distribution and mitigates severe material loss. Figure 4f illustrates the erosion crater of the C_2_B_4_C_2_ sample. Similar to B_2_C_4_B_2_, the erosion is less concentrated in a central region and more evenly spread across the surface. The crater depth is slightly higher than in B_2_C_4_B_2_ but remains shallower than in B_8_ and C_8_. The improved performance of this sample confirms that a balanced basalt–carbon configuration provides better erosion resistance compared to monolithic structures, as it reduces localized stress concentrations and material removal.

**Figure 5:**
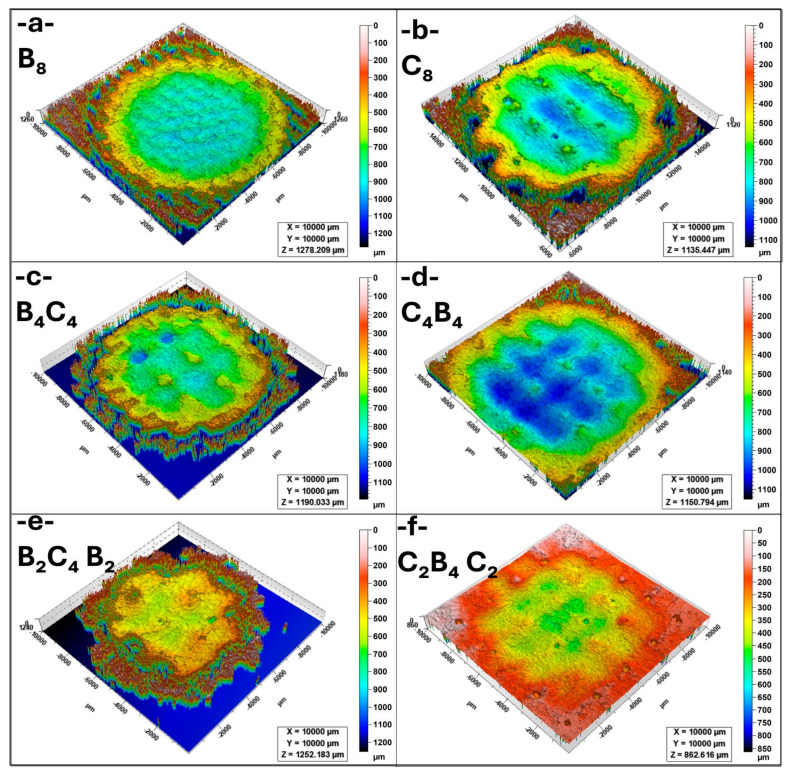
Comparison of 3D images of erosion crater at 90° impingement angle and 80 °C (**a**) B_8_, (**b**) C_8_, (**c**) B_4_C_4_, (**d**) C_4_B_4_, (**e**) B_2_C_4_B_2_, (**f**) C_2_B_4_C_2_.

Figure 5a presents the erosion crater of the B_8_ sample at 80 °C. Compared to Figure 4a (25 °C), the erosion depth is slightly reduced, suggesting that higher temperatures may influence the material’s erosion behavior by altering its fracture mechanics. The crater remains well defined, with a distinct central region of significant material loss. The surrounding edges show material accumulation, indicating material removal via brittle fracture mechanisms. Figure 5b illustrates the erosion crater of the C_8_ sample. The erosion depth appears to be lower than in B_8_, but the overall material removal is still substantial. The surface morphology indicates a more dispersed erosion pattern, likely due to the temperature-induced softening of the carbon layers. Compared to 25 °C (Figure 4b), the erosion characteristics suggest a shift from brittle fracture to a more ductile erosion mechanism. Figure 5c displays the erosion crater of the B_4_C_4_ sample, which shows improved resistance compared to the monolithic B_8_ and C_8_ structures. The crater depth is moderate, and the erosion pattern appears more heterogeneous. The presence of alternating basalt and carbon layers seems to contribute to better energy dissipation, reducing localized material removal. The erosion morphology remains consistent with previous observations, with erosion pits distributed across the surface. Figure 5d shows the erosion crater of the C_4_B_4_ sample. Similar to B_4_C_4_, the crater is shallower than those in B_8_ and C_8_, suggesting improved erosion resistance due to the layered structure. The erosion pattern appears more uniform compared to lower temperatures, possibly due to the stabilization of interfacial interactions between the basalt and carbon layers. The crater edges remain well defined, with no significant signs of excessive material loss. Figure 5e presents the erosion crater of the B_2_C_4_B_2_ sample, which continues to demonstrate enhanced erosion resistance. The crater depth remains moderate, but the erosion pattern is more irregular than in the other composite samples. The presence of multiple small erosion pits suggests that the layered structure effectively mitigates severe material loss by distributing impact stress across the surface. Figure 5f illustrates the erosion crater of the C_2_B_4_C_2_ sample, which exhibits the shallowest erosion depth among all samples. The crater morphology indicates a more uniform material loss, with no distinct central erosion region. This suggests that the basalt–carbon layering provides significant resistance against erosion, particularly at elevated temperatures. The overall reduction in erosion depth further confirms that the C_2_B_4_C_2_ structure is one of the most erosion-resistant configurations under these conditions.

**Figure 6:**
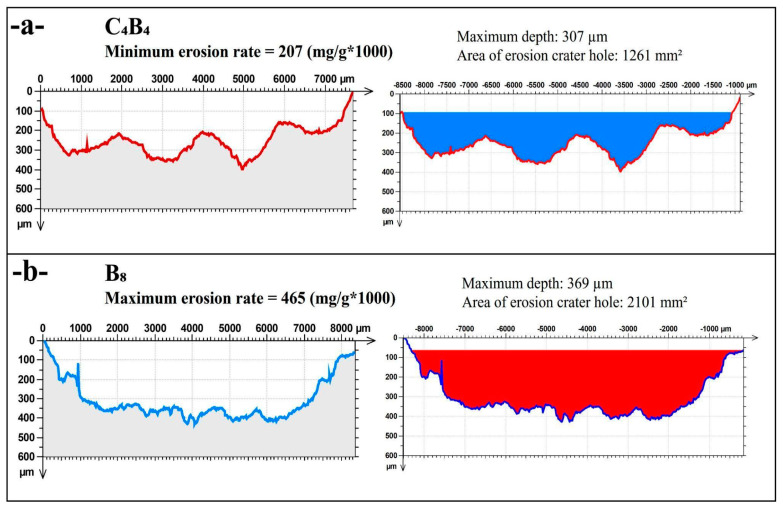
Comparison of erosion crater depth and crater mid-section area at a 90° impingement angle, with maximum and minimum erosion rate values at temperatures of 25 °C and 80 °C: (**a**) C_4_B_4_, (**b**) B_8_.

Figure 6 presents the mean values of the model for the mid-section total area. The factor analysis compares samples tested under a 90° impingement angle within a temperature range of 25 °C to 80 °C. Among these, the highest and lowest amounts of eroded particles were observed. The specimen with the best performance exhibited the lowest erosion rate, although it did not display the same erosion behavior consistently under these conditions. The erosion crater profiles of the C_4_B_4_ sample, which showed the minimum erosion rate, are shown in Figure 6a. The maximum measured depth of the erosion crater is ∼307 µm, with a total crater area of 1261 mm^2^. Unlike the deep cavity in the eastern flank, this pattern is more uniform, with multiple shallow depressions indicating the eroded pattern. This means that the alternating layers of carbon and basalt play a role in absorbing the impact energy more evenly, preventing localized material removal. Thus, the combination of material properties in the C_4_B_4_ configuration likely contributes to its synergistic effect and increases erosion resistance, evidenced by the lower erosion rate and smaller crater dimensions.

Figure 6b presents the profile of the erosion crater of the B_8_ sample, which shows the highest level of erosion. The maximum depth of the erosion crater is 369 µm, where it is about two times deeper than the C_4_B_4_ sample, and the total crater area is up to 2101 mm^2^, indicating that a large volume of material is removed. The erosion profile consists of a deep central zone and steep edges, indicative of the dominant material removal mechanism being brittle fracture. The increased surface exposed with an even larger crater underscores that the monolithic basalt is quite vulnerable to erosive processes, either due to its brittle nature and/or the absence of an alternative accident phase that “reinforces” the initial phase, making it much more likely than the previous volume models to leave behind fewer more fragile plates. As can be seen from the comparison between B_8_ and C_4_B_4_, the composite structures provide a better erosion resistance than the same thickness in a single material.

### 3.3. Damage Mechanisms of Erosion Craters (SEM)

**Figure 7:**
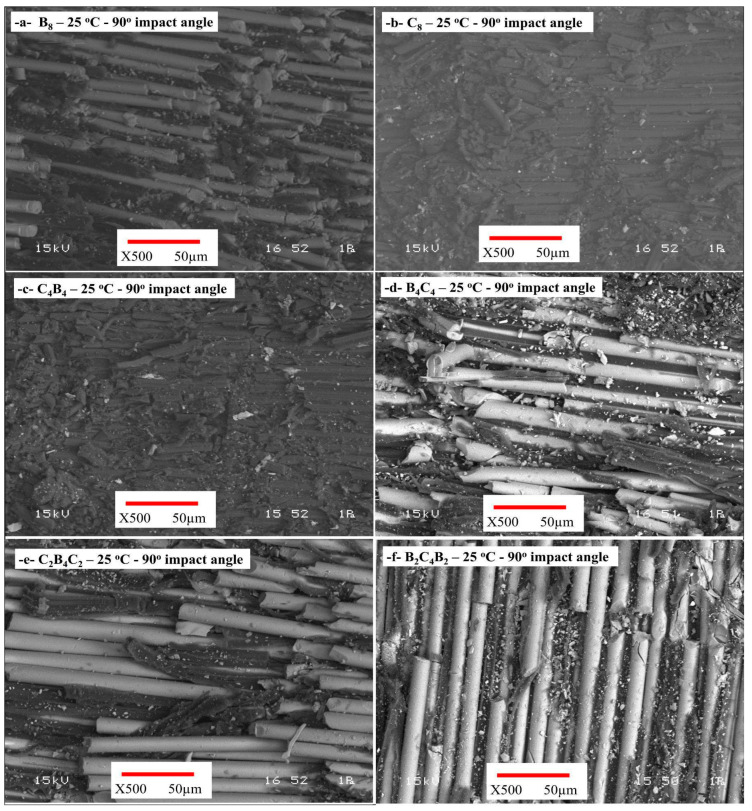
SEM images of the wear damage surface of the samples after 90° wear tests at room temperature: (**a**) B_8_, (**b**) C_8_, (**c**) C_4_B_4_, (**d**) B_4_C_4_, (**e**) C_2_B_4_C_2_, (**f**) B_2_C_4_B_2_.

Figure 7 displays the wear damage surface images of the samples subjected to 90° wear tests conducted at room temperature. Upon examination of the surface images of the sample designated B_8_, a pronounced and substantial wear crater is observed (Figure 7a). Significant material loss was noted at the center of the wear crater, accompanied by pronounced sharp edges surrounding it. This scenario indicates a brittle fracture mechanism resulting from brittleness. Fractures and dislocations in the fibers were identified as the primary damage mechanism. In the C_8_ samples, the carbon fibers exhibited greater dispersion during wear, with linear fractures and matrix tears noted along the fiber orientation (Figure 7b). Despite the material exhibiting more uniform wear, crack propagation was distinctly detected between the fibers subjected to high energy. In the hybrid composite samples designated C_4_B_4_ and B_4_C_4_, the crater exhibited reduced depth, and uniformly distributed micro pits were noted on the surface (Figure 7c,d). Fractures aligned with the fiber orientation restricted crack propagation when the carbon fibers were positioned on the worn surface. The samples coded C_2_B_4_C_2_ and B_2_C_4_B_2_ showed small pit structures and uneven wear morphologies (Figure 7e,f). There were few fiber fractures, and the degradation was primarily seen as matrix cracks. The SEM photographs showed more uniform wear on the surface and a reduction in fibrous damage. There were few, if any, fiber fractures, while matrix deformation was present.

**Figure 8:**
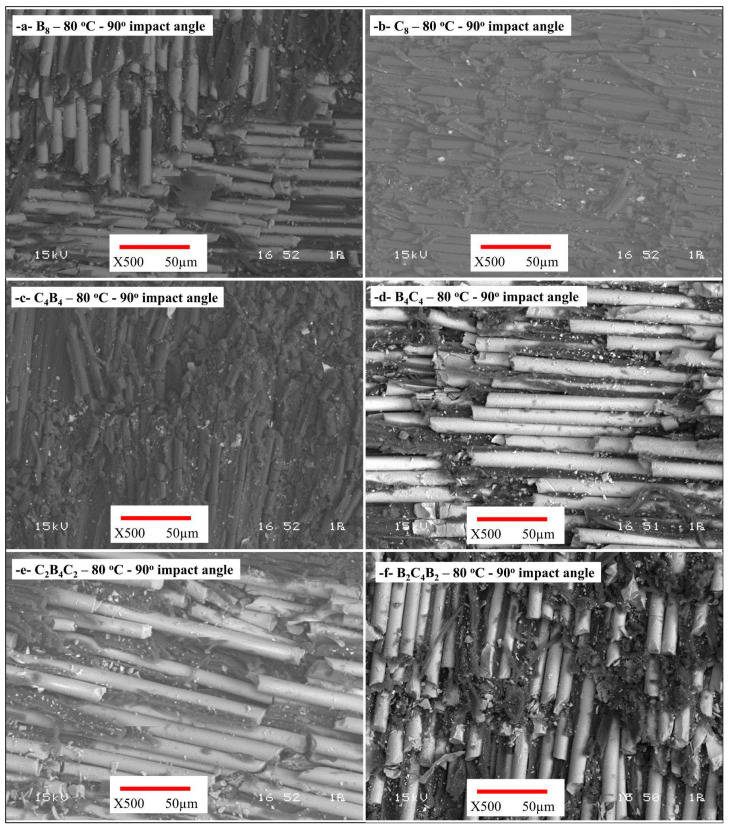
Wear damage surface SEM images of the samples after 90° wear tests at 80 °C temperature (**a**) B_8_, (**b**) C_8_, (**c**) C_4_B_4_, (**d**) B_4_C_4_, (**e**) C_2_B_4_C_2_, (**f**) B_2_C_4_B_2_.

The images of wear damage on the surfaces of the samples after the 90° wear tests performed at 80 °C are shown in Figure 8. Although the wear depth slightly decreased in the B_8_, the crater is still evident (Figure 8a). It is observed that the brittle fracture effect decreases with the increase in temperature, but that the surface deformations become more widespread. Bond cracks and fiber dislocations are remarkable at the fiber–matrix interface. In the C_8_, it was determined that there was less and more irregular brittle behavior, and that superficial wear occurred depending on the glass transition temperature of the matrix (Figure 8b). In the C_4_B_4_-coded sample, the wear behavior is more uniform (Figure 8c). Fiber breaks are fewer, and matrix deformations are more widespread. In the B_4_C_4_ sample, the crater depth is at a medium level. It was observed that the fiber–matrix fit was better, and that local deformations occurred depending on the temperature (Figure 8d). The fibers generally remained in place, and the breaks were reduced. In the samples coded C_2_B_4_C_2_ and B_2_C_4_B_2_, the crater depth decreased significantly (Figure 8e,f). The fibers generally remained in place and prevented crack propagation depending on the glass transition temperature of the matrix. Instead of fiber ruptures, deformations around the fibers were common. The arrangement of the composite internal structure directly affects the wear resistance. In particular, as in the C_2_B_4_C_2_ and B_2_C_4_B_2_ samples, the balanced layer arrangement of carbon and basalt significantly reduced wear. It was observed that these structures formed lower depth craters and fewer fiber fractures.

### 3.4. Factorial Design and Analysis of Variance (ANOVA)

The factorial design approach is a statistical method used to analyze the impact of numerous elements and their interactions in a structured manner. Factorial experimental designs are widely used in the literature, particularly in engineering and materials science, to report the individual and synergy effects of various process factors on outcomes. The approach enables the examination of both the main and the interaction effects of the constituents. As a result, an exhaustive assessment of process optimization, performance prediction, and variable effect determination can be established. The ANOVA analysis shown in Table 1 was performed using a factorial experimental design. Upon examination of the table, it is evident that the model exhibits no total error (Error DF = 0), indicating that the model was constructed to include all variables, and that all variables were really incorporated into the model. The total sum of squares (Seq SS) for the model is 518,709, representing 100% of this value. The examined variables include Material, Temperature, and Impingement Angles. These are regarded as linear inside the model and possess a cumulative total of nine degrees of freedom (DF). Linear effects account for 63.66% of the overall variability. The material component has five degrees of freedom and accounts for 43.17% of the overall variability. This indicates that the kind of material is a significant determinant of the outcomes. The temperature variable has three degrees of freedom, and its impact on the model is quantified at 19.97%. The impact angle has one degree of freedom and accounts for just 0.52% of the overall variability, suggesting that it is less influential than the other factors.

Table 2 shows the ANOVA analysis of solid particle erosion rate. The two-way interactions, which look at the interaction between two components, have 23 degrees of freedom and account for 28.82% of the overall variability. Material–temperature interaction accounts for 9.91%, whereas material–impact angle interaction contributes more significantly at 18.22%. The temperature–impact angle interaction accounts for 0.69% of the overall variability. These values indicate that there is some interaction in some of the parameters when paired. The three-way interactions, in which three components are considered simultaneously, possess 15 degrees of freedom and contribute 7.51% of the overall variability. The Material, Temperature, and Impingement Angles interaction accounts for the whole of this contribution. This example demonstrates that there is a degree of interaction in the three components when taken together. The factorial ANOVA analysis reveals that material type is the dominant component, with 43.17%. The material and temperature interaction contributes 9.91%, while the material and impact angle interaction take a bigger 18.22%. Although the triple interactions are a small proportion of the overall variability (7.51%), it is a valuable discovery in examining the overall interactions of the system. The absence of total error in the model indicates the components to be under fixed control and, as such, the explanatory power is high. Factorial experimental designs, as such, have widespread use in material studies, process optimization, and in engineering. This study is critical in determining the effect of many elements and their interactions and results in more informed and data-driven outcomes in decision-making.

**Figure 9:**
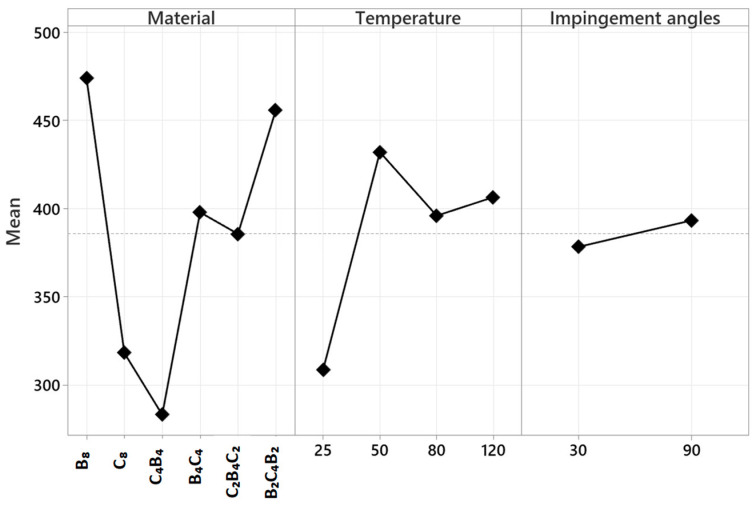
Main effect plot graph of solid particle erosion test performed using a garnet abrasive according to the material type, temperature and impact angle on erosion rate.

Figure 9 illustrates the primary effect plot graph displaying the outcomes of the solid particle erosion test using garnet abrasive. The mean values shown on the Y-axis indicate the erosion rate in mg/g * 1000. A high erosion rate indicates that the material exhibits more erosion (indicating poor wear resistance), while a low erosion rate indicates that the material has enhanced wear resistance. When evaluated in terms of material variable, B8 has the highest erosion rate and has the highest material loss. C_8_ has the lowest erosion rate and is the structure most resistant to abrasion. The C_4_B_4_, B_4_C_4_, C_2_B_4_C_2_ and B_2_C_4_B_2_ composites are at an intermediate level. These results show that composites with a higher carbon fiber content have higher abrasion resistance. As the basalt content increases, the erosion rate also increases, meaning that the material wears more. This may be due to the fact that basalt fibers are hard but brittle and lose material more quickly against high-energy particle impacts. On the other hand, the high toughness of carbon fibers may cause them to absorb the energy of the impacting particles better and thus have a lower erosion rate.

The effect of temperature on the erosion rate is related to the mechanical response of the polymer matrix and the deformation mechanisms that are changed with temperature. The toughening of the material at 50 °C can make the polymer matrix more ductile and capable of absorbing the impact energy. Since the polymer matrix can be harder and brittle at room temperature (25 °C), the brittleness effect increases during solid particle impacts and can accelerate the wear. However, the toughening of the matrix at 50 °C can reduce the crack formation and surface fractures caused by particle impact. In addition, at high temperatures, the chain mobility increases, the energy of the impacting particles is absorbed more homogeneously and local fractures are reduced, resulting in lower erosion rates. However, the increase in the wear rate again at 80 °C and 120 °C is related to the polymer matrix approaching or exceeding the glass transition temperature. At these temperatures, the matrix softens and loses its hardness, the bonds weaken due to thermal oxidation and the matrix–fiber interface is damaged, causing the fibers to break more easily. In addition, at higher temperatures, the polymer matrix becomes less resistant to solid particle impacts and the plastic deformation rate on the surface increases. When evaluated in terms of material composition, it was observed that increasing the carbon fiber content generally increases the wear resistance, while basalt fiber exhibits lower wear resistance. In particular, the highest erosion rate of sample 8B suggests that basalt fiber is not as wear resistant as carbon fiber. It is seen that the wear rate decreases with increasing carbon fiber content, but the fiber arrangement and matrix–fiber interface also play an important role in this process. In conclusion, the effect of temperature on the erosion resistance is not linear; while the material performance improves in certain temperature ranges, it deteriorates at extreme temperatures. Therefore, the operating temperature is a critical factor related to the use of polymer matrix composites in corrosive environments, and the thermal stability of the matrix material should be optimized to increase wear resistance at high temperatures.

When evaluated in terms of the impact angle variable, the highest erosion rate was observed at the 30° impact angle. In contrast, the erosion rate decreases at the 90° impact angle, meaning that the material becomes more durable. This trend is significant when evaluated in terms of erosion mechanisms. At shallow impact angles (30°), the cutting mechanism dominates, i.e., the impinging particles scrape and erode the surface, and thus the erosion is high. At a 90° impact angle, where the particles impact the surface normally, the plastic deformation mechanism dominates and some of the energy gets absorbed into the material, which reduces material loss. The results show that the cutting mechanism dominates at oblique angles and the deformation mechanism dominates at right angles in polymer matrix composites.

In general, with an increasing content of carbon fiber, the erosion rate decreases; thus, the material is more resistant to wear. With an increasing content of basalt, the erosion rate increases; thus, the material wears more. Although maximum wear is at 25 °C, the lowest erosion rate is achieved at 50 °C. At higher temperatures (80 °C and 120 °C), the wear rate increases again. While maximum erosion is observed at the 30° impact angle, the wear resistance increases at the 90° angle. These findings indicate that design parameters should be optimized to improve the performance of the composite materials used in the aerospace, automotive and energy sectors in corrosive environments. In particular, increasing the carbon fiber content and carefully controlling the operating temperatures can increase erosion resistance.

## 4. Conclusions

In this research, the high-temperature solid particle erosion behaviors of basalt–carbon fiber hybrid composites with various ply sequences (B_8_, C_8_, C_4_B_4_, B_4_C_4_, C_2_B_4_C_2_, B_2_C_4_B_2_) were thoroughly investigated using garnet abrasive particles under different temperatures (25 °C, 50 °C, 80 °C, and 120 °C) and impingement angles (30° and 90°). The experimental data revealed substantial variations in the erosion rate based on the composite configurations, test temperatures, and particle angles of impact. Among the studied specimens, monolithic basalt (B_8_) and carbon (C_8_) composites showed the highest erosion rates, especially at high temperatures, with a maximum of 544.9 mg/g (120 °C, 30°) for B_8_ and a maximum of 541.3 mg/g (120 °C, 90°) for C_8_. This result emphasizes the susceptibility of single-fiber composites to severe erosive damage at higher temperatures, associated with an increased brittleness in basalt and structural deterioration in carbon-based composites. In comparison, hybrid laminate composites showed a considerable enhancement in erosion resistance with the positive synergistic benefits of hybridization. In these conditions, the lowest erosion rate was 200 mg/g for the C_4_B_4_ composite, which showed excellent erosion resistance. Nevertheless, it must be noted that the erosion behavior of the C_8_ arrangement also showed a similar erosion resistance for certain conditions (e.g., 176.9 mg/g for 25 °C, 30°), which proved that carbon-rich or carbon-outward layups play a protective role in preventing wear. In addition, laser-profilometry-aided crater analysis confirmed shallower crater depths for hybrid composites compared to single-component assemblies, highlighting once again the enhanced protection of well-balanced basalt–carbon fiber composites.

Statistical analysis by factorial analysis of variance revealed that material composition (43.17%) influenced erosion the most; this was seconded by temperature (19.97%) and, to a lesser extent, impingement angle (0.52%). Material–temperature (9.91%) and material–impact angle (18.22%) interactions were significant factors controlling the erosion behavior, highlighting the multidimensional character of erosion behavior under various contexts.

SEM analysis confirmed the dominant wear mechanisms, which indicated that hybrid composites suppressed extensive fiber breakage as well as matrix cracking due to the effective dissipation of energy through composite layers. The integrity of the fiber–matrix interface in hybrids enhanced abrasion resistance over single-type fiber composites by lowering local stress concentrations, with improved wear profiles being more homogeneous.

There is a need to further optimize ply sequences and find other fiber matrix alterations, including surface treatments or the integration of nano-scale fillers, to improve composite resistance against extreme erosion and heat exposure. Thorough numerical modeling as well as predictive simulations using machine learning approaches can also be used to predict erosion performance effectively and enable the development of more durable composite materials for aerospace, automobile, and renewable energy applications. More sophisticated techniques, including response surface methodology (RSM), genetic algorithms, or machine learning models, will be used in future research to further refine process parameters and build predictive models using significant variables derived from current studies.

## Figures and Tables

**Table 1:** Erosion rates of the composites depending on temperature and impingement angles.

Exp. No	Sequence	Temperature (°C)	Impingement Angles (°)	Erosion Rate (mg/g * 1000)	Exp. No	Sequence	Temperature (°C)	Impingement Angles (°)	Erosion Rate (mg/g * 1000)
1	B_8_	25	30	440.4	25	B_4_C_4_	25	30	343.6
2	B_8_	25	90	432.4	26	B_4_C_4_	25	90	283.1
3	B_8_	50	30	477.3	27	B_4_C_4_	50	30	481.3
4	B_8_	50	90	531.1	28	B_4_C_4_	50	90	380.4
5	B_8_	80	30	501.0	29	B_4_C_4_	80	30	461.0
6	B_8_	80	90	464.9	30	B_4_C_4_	80	90	458.7
7	B_8_	120	30	544.9	31	B_4_C_4_	120	30	433.3
8	B_8_	120	90	400.0	32	B_4_C_4_	120	90	341.0
9	C_8_	25	30	176.9	33	C_2_B_4_C_2_	25	30	233.3
10	C_8_	25	90	284.0	34	C_2_B_4_C_2_	25	90	319.1
11	C_8_	50	30	282.7	35	C_2_B_4_C_2_	50	30	383.1
12	C_8_	50	90	428.9	36	C_2_B_4_C_2_	50	90	531.1
13	C_8_	80	30	205.3	37	C_2_B_4_C_2_	80	30	392.4
14	C_8_	80	90	372.0	38	C_2_B_4_C_2_	80	90	410.0
15	C_8_	120	30	252.9	39	C_2_B_4_C_2_	120	30	368.9
16	C_8_	120	90	541.3	40	C_2_B_4_C_2_	120	90	446.2
17	C_4_B_4_	25	30	200.0	41	B_2_C_4_B_2_	25	30	373.8
18	C_4_B_4_	25	90	207.1	42	B_2_C_4_B_2_	25	90	408.4
19	C_4_B_4_	50	30	284.9	43	B_2_C_4_B_2_	50	30	576.9
20	C_4_B_4_	50	90	265.8	44	B_2_C_4_B_2_	50	90	560.4
21	C_4_B_4_	80	30	308.0	45	B_2_C_4_B_2_	80	30	491.6
22	C_4_B_4_	80	90	310.0	46	B_2_C_4_B_2_	80	90	375.6
23	C_4_B_4_	120	30	336.4	47	B_2_C_4_B_2_	120	30	526.2
24	C_4_B_4_	120	90	352.0	48	B_2_C_4_B_2_	120	90	333.8

**Table 2:** ANOVA analysis of solid particle erosion rate.

Source	DF	Seq SS	Contribution	Adj SS	Adj MS
Model	47	518.709	100.00%	518.709	11.036
Linear	9	330.234	63.66%	330.234	36.693
Material	5	223.916	43.17%	223.916	44.783
Temperature	3	103.599	19.97%	103.599	34.533
Impingement angles	1	2718	0.52%	2718	2718
2-Way Interactions	23	149.506	28.82%	149.506	6500
Material × Temperature	15	51.382	9.91%	51.382	3425
Material × Impingement angles	5	94.531	18.22%	94.531	18.906
Temperature × Impingement angles	3	3593	0.69%	3593	1198
3-Way Interactions	15	38.969	7.51%	38.969	2598
Material × Temperature × Impingement angles	15	38.969	7.51%	38.969	2598
Error	0	0	0	0	0
Total	47	518.709	100.00%		

## Data Availability

The datasets presented in this article are not readily available because the data are part of an ongoing study. Requests to access the datasets should be directed to Corresponding Author.

## Abbreviations

The following abbreviations are used in this manuscript:

FRP	Fiber-reinforced polymer
SEM	Scanning electron microscope
ANOVA	Analysis of Variance
